# A mixed methods pilot feasibility study of the unified protocol group psychotherapy for early bipolar disorder

**DOI:** 10.3389/fpsyt.2025.1524243

**Published:** 2025-05-27

**Authors:** Magnus Johan Engen, Siv Hege Lyngstad, Erlend Eikenæs, Jon Vøllestad, Margrethe Collier Høegh, Trine Vik Lagerberg, Sofie Ragnhild Aminoff

**Affiliations:** ^1^ Nydalen District Psychiatric Center, Division of Mental Health and Addiction, Oslo University Hospital, Oslo, Norway; ^2^ Department of Clinical Psychology, Faculty of Psychology, University of Bergen, Bergen, Vestland, Norway; ^3^ Section for Psychosis Research, Division of Mental Health and Addiction, Oslo University Hospital, Oslo, Norway; ^4^ Department of Research and Development, Division of Mental Health and Addiction, Oslo University Hospital, Oslo, Norway; ^5^ Department of Psychology, Faculty of Social Sciences, University of Oslo, Oslo, Norway; ^6^ Early Intervention in Psychosis Advisory Unit for South East Norway, Division of Mental Health and Addiction, Oslo University Hospital, Oslo, Norway

**Keywords:** unified protocol (UP), bipolar disorder, feasibility & acceptability, affect regulation, emotion regulation, mixed methods

## Abstract

In this pilot study we explored the feasibility and acceptability of “The Unified Protocol for Transdiagnostic Treatment of Emotional Disorders” (UP) in a group format for individuals with early bipolar disorder (BD). Using a mixed methods design we integrated quantitative assessments and qualitative interviews to examine the practical application of UP in a clinical setting with a focus on how participants experienced the treatment. Nine participants with BD diagnoses completed the 12-session group intervention with modules focusing on emotion regulation through skills in non-judgmental awareness, cognitive flexibility, and exposure strategies. Quantitative findings indicated significant improvements in affective lability, overall functioning, and well-being. Qualitative findings highlighted participants’ appreciation for the structured format and peer support from the group, which facilitated exploration of emotional experiences and skills practice. Participants reported particular benefit from non-judgmental awareness exercises, notably the “three-point check,” and valued the group’s role in reducing isolation and promoting shared learning. The study underscores UP’s potential adaptability for individuals with BD in a group format. Possible adjustments are discussed, such as strengthening the focus on affective symptoms as well as increasing session duration and customizing exposure exercises for BD-specific challenges. Overall, the UP group format shows promise as a feasible, acceptable, and potentially effective adjunct treatment for BD, with room for targeted refinements to optimize outcomes.

## 1 Introduction

Bipolar disorders (BD) make up a spectrum of affective disorders impacting 2.4% of the world population and is among the main causes of disability in young people (1). Anxiety and other psychiatric comorbidities are frequent and influence the course of illness (2, 3). Evidence favors psychotherapy as an adjunct to medication in the maintenance of euthymia (4), but a growing evidence base also demonstrates how psychotherapy may be particularly indicated for comorbid anxiety in BD (2, 5, 6).

More recently, the role of childhood trauma, such as emotional abuse and neglect, and high levels of affective lability (both in mood episodes and in euthymia) are gaining attention as important areas for targeted treatment (7, 8). Affective lability, defined as the propensity to experience rapid, unpredictable and excessive changes in affect (9), is recognized as a promising transdiagnostic treatment target that is also shown to be highly prevalent across the bipolar spectrum (10, 11). The existing weight of evidence from psychotherapy in BD comes mainly from diagnosis-specific treatments emphasizing skill-building and psychoeducation where the main goal is reduction in episode recurrence (12). However, the complexity of the adversities associated with BD calls for a broader focus in treatment models, especially for the large group of patients with significant psychiatric comorbidities (6).

The Unified Protocol (UP) (13, 14) is an evidence-based treatment developed to target difficulties with emotion regulation. Instead of addressing specific diagnoses, the UP is aimed at the broader construct of emotional disorders characterized by frequent negative emotions that the individual reacts aversively to and tries to manage through avoidant strategies. This enables clinicians to approach different and co-occurring clinical presentations using a coherent framework which emphasizes their underlying similarities (15). Because BD is a complex disorder where frequent comorbidities and affective dysregulation may play a crucial role in shaping the course of illness, the UP has been proposed as a particularly well-suited treatment for this population. Many individuals with BD struggle with managing their emotions, and have patterns of negative reactions and maladaptive regulation attempts that align well with the functional model of emotional disorders that the UP is based upon (16, 17). One randomized feasibility trial has been piloted with the UP for BD showing promising results (18). This study recruited patients with BD and comorbid anxiety disorders and the treatment format was individual therapy (18). The strongest evidence for the UP is from the individual format where it has shown to be non-inferior to diagnosis-specific CBT (14). However, a growing evidence base supports the application of the group format which has been found to be both superior to “treatment as usual” (19) and non-inferior to group-based diagnosis-specific CBT (20). The group format is extensively applied in the maintenance treatment of BD with group psychoeducation for which there is good evidence (21, 22). Moreover, a review by Miklowitz et al. further showed that psychoeducation with guided practice of illness management skills in a family or group format was superior to the individual format in reducing recurrence of mood episodes (4). Because the UP with its transdiagnostic focus goes beyond the BD-specific elements in psychoeducation and is widely applicable for common comorbidities, the implementation of the UP may play an important role in making evidence based psychological treatment more accessible. The cost-effectiveness of the group format in UP makes it more feasible to implement in health services where resources are limited (23). Furthermore, as individuals with BD frequently struggle with self-stigma (24), group processes promoting health through normalization, shared experiences, and peer-assisted learning are relevant beyond the specific ingredients in the UP (25).

The goal of the current study was to explore the use of the UP in a group format for improving affect regulation and anxiety tolerance in a naturalistic sample of early course BD. Using both qualitative interviews and quantitative measures, we addressed the following questions:

- How did the participants with BD experience the UP treatment, particularly regarding the group format?
- What were the specific challenges and difficulties with the treatment reported by the participants?
- Was the intervention acceptable?
- Was the intervention feasible with regards to recruitment and retention rates?

## 2 Materials and methods

### 2.1 Study design

The study was designed to explore pre- and post-intervention measures, as well as variables relevant for feasibility (i.e. drop-out rates etc.). The study had a mixed methods pilot feasibility study design, where quantitative and qualitative approaches were integrated to examine the acceptability and feasibility of the UP group intervention for BD (26).

### 2.2 Ethics

All study participants took part in pre-intervention interviews where relevant details about the intervention and study were presented. All study participants provided written informed consent prior to their participation. The study was approved by the regional ethical committee (Application ID #701148).

### 2.3 Participants

All participants were recruited from a specialized treatment unit for early BD. Early BD was defined as having received the diagnosis of BD for the first time within the last year. The patients were typically referred by general practitioners to secondary public healthcare services at Oslo University Hospital (OUS), Nydalen Psychiatric Center. The center serves a catchment area of approximately 135.000 inhabitants in Oslo. Diagnoses were based on the Structured Clinical Interview for DSM-5 Clinical Version (27) and determined by trained professionals at the early BD unit who had gone through a formal training program. The diagnoses are also discussed in weekly diagnostic consensus meetings with senior psychiatrists and/or clinical psychologists.

Participants recruited to the study were currently under treatment at the early BD unit having a) a BD-spectrum disorder and b) difficulties with regulating affect and/or anxiety with behavioral avoidance tendencies in euthymic periods. Eligibility for participation was determined by clinical assessments conducted by treating clinicians and reviewed during weekly meetings of the specialized treatment unit for early BD. Additionally, a two-hour individual interview was held before the first group session to assess whether the treatment model aligned with the participants’ presenting issues. All participants had to be in the age range of 18 and 65 years and speak a Scandinavian language. Exclusion criteria were current suicidal ideation or -psychosis, or ongoing substance-use disorder that would interfere with the treatment. All but one participant had completed the 12-session group psychoeducation (PE) program. The participant who had not completed group PE had received individual PE, and all participants had individualized BD relapse prevention plans.

Ten patients were invited to participate in the study. One patient was not able due to BD episode, giving a group total of 9 participants recruited to take part in the study. The mean age of the participants was 28.6 (SD 8.1). Three participants (33%) were male, and six (67%) females. Two had BD type I (22%), six (67%) had BD type II, and one (11%) had BD not otherwise specified. Three participants had no comorbid diagnoses, whereas the remaining six had an average of three diagnoses. Comorbid diagnoses were attention deficit disorder (ADHD) (3/9, 33%), post-traumatic stress disorder (PTSD) (2/9, 22%), obsessive-compulsive disorder (OCD) (2/9 22%), panic disorder (2/9, 22%), social phobia (1/9, 11%), generalized anxiety disorder (GAD) (1/9, 11%) and cannabis use disorder (1/9, 11%).

### 2.4 Procedure

The intervention was conducted in-person from March to May 2024 at Nydalen District Psychiatric Center, OUS. Prior to the group treatment, all participants completed an individual 2-hour interview based on module 1 in the treatment manual. This interview was used to create an individual case formulation, a treatment plan with specific steps, and to map down pros and cons of committing to change. It also prepared participants for the first group session and provided an opportunity to withdraw if the treatment model did not seem like a good fit.

#### 2.4.1 The unified protocol for transdiagnostic treatment of emotional disorders group psychotherapy intervention

The treatment consisted of 12 weekly 2-hour sessions following principles described in the treatment manuals (13, 14). Two specialists in clinical psychology (M.J.E and E.E.) and one psychiatrist (S.H.L.) led the group intervention. All therapists have extensive experience working with psychotherapy and patients with BD. Before the intervention, the therapists had received formal training in the UP in a two-day workshop led by certified UP therapists from the Faculty of Psychology, University of Bergen, and through studying the UP manual (13, 14). All sessions were videotaped for supervision. The therapists were rated for treatment fidelity after all sessions to ensure adequate levels of adherence and competence in the treatment model. Supervision and ratings were provided by a clinical psychologist (J.V.) who is certified as therapist and trainer in the UP group format. All three therapists were rated as showing satisfactory competence and adherence to the UP (score ≥ 80% based on the Adherence Rating Scale for UP).

The treatment modules administered over the 12 sessions are presented in **Table 1**. All sessions from module 2 onwards started with group members sharing experience with previous homework and ended with preparing new homework assignments. As a tool for tracking depression and anxiety, participants completed the Overall Depression Severity and Impairment Scale (ODSIS) (28) and the Overall Anxiety Severity and Impairment Scale (OASIS) (29) before each session and were paired with a fellow group participant to discuss their ODSIS and OASIS scores. Written handout material corresponding to the UP Patient Workbook (14) was used in all sessions to facilitate understanding of concepts, learning of skills, and completion of homework assignments. In Module 1, the initial focus was on the group members to present themselves and their goals for the treatment. This was followed by psychoeducation about the importance of motivation and group activities. Module 2 focuses on the nature of emotions and how they are meant to serve an adaptive function. Participants were introduced to a framework of emotions as constituted by the three interacting components of thoughts, impulses/behaviors and bodily sensations, and practiced identifying triggers for emotional experiences as well as short- and long-term consequences of their reactions to them. Module 3 introduces a non-judgmental attitude towards emotional experience to counteract the reactive and avoidant patterns characterizing emotional disorders. In this session the participants were invited to explore different emotional states using guided mindfulness practices. A brief mindfulness practice called the three-point-check was introduced, enabling participants to relate to their emotions in daily life with a present-centred and non-judgmental attitude. Modules 4, 5 and 6 focuses on each of the three components of emotional experience separately. Module 4 provides skills to identify rigid and automatic cognitive appraisals, and to facilitate flexible thinking instead. Module 5 focuses on emotion-driven behavioral tendencies and behavioral avoidance in the face of strong or unwanted emotion. Participants were presented with examples of typically driven or avoidant behaviors. They were then given handouts to specify their own behaviors and prepare opposite actions as homework assignments. In Module 6, the focus is on recognizing and confronting difficult bodily sensations, with interoceptive exposure exercises to increase tolerance for such sensations. Reactions were discussed and reflected upon in a group process. Module 7 comprises 5 sessions which are all focused on participants implementing their newly acquired skills to conduct emotional exposures in line with their personal treatment goals. The participants were exposed to both internal and external emotional triggers through a wide range of tasks that was carried out both inside and outside of the treatment facility. Role-play, speaking in front of a group and entering public situations were some of the exposures used by the participants to address their difficulties. The three therapists divided the group according to the needs of the participants to ensure maximum level of effort and engagement. Two separate rooms were available for exposures. Participants also made use of public places outside the hospital. In Module 8 the focus is on lessons learned, how to consolidate and maintain gains from treatment, and how to further implement what they found useful from the group to prevent relapse.

**Table 1:** Content of intervention modules.

Module	Agenda	Session
**1**	**Goal setting and motivation** Establishing UP case conceptualization and treatment goals, and enhancing motivation by exploring benefits and costs of change	1
**2**	**Understanding emotions** Explaining the adaptive function of emotions and their three constituent components (thoughts, physical sensations and behaviors), as well as the temporal unfolding of emotions in terms of antecedents, responses and consequences (“The ARC of emotions)	2
**3**	**Mindful emotion awareness** Developing the capacity for non-judgmental and present-centred awareness of emotional responses	3
**4**	**Cognitive flexibility** Understanding the importance of thoughts in emotional experience, and developing ways of relating more flexibly to common negative automatic appraisals (“thinking traps”).	4
**5**	**Countering emotional behaviors** Exploring the range of avoidance strategies and emotion-driven behaviors, and finding alternative actions contrary to emotional urges.	5
**6**	**Understanding and confronting physical sensations** Conducting interoceptive exposure to increase tolerance of uncomfortable physical sensations	6
**7**	**Emotion exposure** Designing and conducting exposure to feared emotions to build tolerance and reduce avoidance	7-11
**8**	**Relapse prevention** Developing a personalized plan to maintain progress and manage future challenges	12

#### 2.4.2 Qualitative assessment - interviews

The interviews with the participants were conducted by two PhDs in clinical psychology (S.R.A. and M.C.H.). Interviewers were not therapists in the intervention and had no prior relation to the participants. All interviews were conducted within 2 weeks of the last treatment session. A semi structured interview guide with open questions was used flexibly. Questions broadly probed for how participants experienced the intervention.

#### 2.4.3 Quantitative assessment

Quantitative data was collected from participants both within two weeks of the first session and right after the last session. The measures were administered by the therapists when information about the intervention was provided along with the informed written consent. With the exception of a clinician-rated measure of manic symptoms, all measures were based on self-report.

##### 2.4.3.1 Quantitative measures

Except for the clinician rated interview for manic symptoms (baseline only) and the self-report measure for satisfaction (post-intervention only), all the measures were repeated at baseline and post-intervention follow-up. Manic symptoms were rated using the clinician-rated interview Bech-Rafaelsen Mania Scale (BRMS). The BRMS consists of 11 items rated from 0-4 with a total range from 0-44, and higher scores reflect more severe states of mania. The scale is extensively used and is shown to have good psychometric properties (30). The BMRS mean score at baseline (N = 9) was 2.11 (SD 2.8, range 0-7).

The following self-report were used: The Quick Inventory of Depressive Symptomatology (QIDS-SR) was used to rate depressive symptoms (31, 32). The QIDS-SR ranges from 0-26, with scores below 5 indicating no depression, 6-10 mild depression, 11-15 moderate depression, 16-20 severe depression, and scores above 21 suggesting very severe depression (32). The 7-item Generalized Anxiety Disorders Scale (GAD-7) was used to assess symptoms of anxiety. Items are scored on a four-point Likert scale (0–3) with total scores ranging from 0 to 21 with scores from 5-9 indicating mild, 10-14 moderate, and above 15 severe anxiety (33). Affective lability was measured using the Affective Lability Scale Short Form (ALS-SF) which consists of 18 items measuring the propensity to experience rapid, unpredictable and excessive changes in affective states. Each item is scored on a 3-point Likert scale (0-3) yielding a total score of affective lability (the sum of responses on all items divided by 18), as well as subscores for three affective domains. Higher scores reflect higher affective lability. The scale has shown good psychometric properties in a Norwegian cohort of participants with BD (34). Emotion regulation was measured using the Difficulties in Emotion Regulation Scale-16 (DERS-16). DERS-16 consists of 16 items measuring the ability to regulate emotions. This entails perceived control of which emotion they have and when, as well as degree of experience and expression. The items are scored on a Likert scale from 0-4, resulting in a total range between 0-64. The DERS-16 has been validated in a Norwegian population (35). The Clinical Outcomes in Routine Evaluation-Outcome Measure (CORE-OM) contains 34 items assessing well-being, symptoms, functioning and risk of harm, producing an overall global distress (GD) score. Items are rated on a 5-point Likert scale (0-4) resulting in a raw GD score from 0-136 which is converted to a mean after dividing by the number of items (36). The World Health Organization Well-Being Index (WHO-5) was included as an additional measure of well-being. All items on the WHO-5 are rated on a 6-point Likert scale (0-5) with total scores ranging from 0-25 with higher scores reflecting greater well-being. Group average scores from 12.5 and below indicate low well-being (37).

The Client Satisfaction Questionnaire (CSQ-8) was used to measure satisfaction with the intervention post-treatment only. The CSQ-8 consists of eight items scored on a four-point Likert scale (0-3) with total scores ranging from 0-24 where higher scores reflect a higher degree of satisfaction (38).

### 2.5 Quantitative analysis

All quantitative analyses were performed using IBM SPSS Statistics for Windows. The Wilcoxon signed rank test was performed on all analyses comparing pre- and post- intervention measures. Significance level for rejecting the null hypothesis was set to < .05 and effect sizes are reported as Pearson’s correlation coefficients *r.*

### 2.6 Qualitative analysis

Our methodological approach for analyzing the qualitative data was thematic analysis (TA) (39). This is a framework for analysis where patterns of meaning are seen as established by the researchers’ interaction with the material in a constructive process. This requires reflexivity on the part of researchers on how their own assumptions, expectations, values and interests may influence the meaning generation process. The epistemological stance in our approach is tempered realism. In our analysis we coded for statements at the surface level of meaning, and themes were developed by induction, driven by the coded data material. The focus of our analysis in this study was on units of meaning that specifically addressed how patients perceived the usefulness of the intervention. Adverse experiences, perceived (ir)relevance of techniques, and the group format for BD using the UP has not yet been tested.

All transcriptions and translations were done by M.J.E, assisted by Whisper Transcription version 10.6.1. Initial coding of the first interview was done by a group of four (S.R.A., M.C.H., M.J.E. and S.H.L.). After collectively agreeing on coding style, M.J.E. who had both listened to and read the interview material several times, coded all interviews. The complete coded material was then discussed by the group to reach a consensus on the broad topics and tendencies in the coded material. Thereafter, the iterative process of generating themes to fit the research questions was done by M.J.E. In this process, the quotes were reread with relation to codes and themes several times.

### 2.7 Mixed analysis

Following separate analyses of the quantitative and qualitative data, the results were reviewed to find an appropriate strategy for using a mixed methods approach. This was done for the three domains: feasibility, acceptability and effect of treatment. In line with recommendations (26), we first evaluated and decided whether all research questions were suited for mixed analysis. We decided that no qualitative interview data meaningfully addressed the feasibility domain in a way that was clearly distinct from acceptability or effect of treatment. For the acceptability domain, two of the main qualitative themes were suited, as well as the CSQ-8. All pre- and post-intervention analyses were found to be related to the effect of treatment. Further, the effect of treatment was related to one of the three main qualitative themes. To ensure anonymity of participants, their statements are not presented with pseudonyms that can link them together. However, care was taken to present statements from all participants, and that no participant is quoted more than once to highlight the same point.

## 3 Results

### 3.1 Feasibility

All participants completed the intervention resulting in an attrition rate of 0%. The average attendance was 10.4 (SD 1.3) out of 12 sessions (87%). Two participants who were voluntarily hospitalized due to depression during the treatment completed from hospital admission by being granted leave of absence for treatment sessions. Two participants who experienced hypomanic episodes during the intervention also agreed to complete the treatment as it was not evaluated to disrupt the group process.

### 3.2 Quantitative results

The comparison between pre- and post-intervention measures showed significant findings for reduction in affective lability (ALS-SF), increased level of functioning (CORE Functioning), and increased well-being on two separate measures (WHO-5 and CORE Well-being) (**Table 2**). All non-significant changes in group averages were in the expected direction of reduced symptoms and problems. The effect sizes for all significant findings were large (*r* = 0.74 to *r* = 0.89). Due to severe depressive symptoms, one participant was not able to complete the post-intervention quantitative outcome measures apart from the CSQ-8.

**Table 2:** Joint display of results.

Feasibility		
Topic or (sub)theme	Quantitative support	Qualitative support
Attendance	Attrition rate 0%. Average attendace 10.4 (1.3) out of 12 sessions, 87%.	No relevant statements
Acceptability		
Topic or (sub)theme	Quantitative support	Qualitative support
Group format	CSQ-8 (*n* = 9) 28.78 (1.99)	Yes: *I'm very glad that I've been in a group. It's really comforting not to feel alone. And it's also good to be in such a challenging situation where others understand*
Relevance		Yes: *It was understandable. We went through it very systematically* No: *That list they had made with suggestions for exposures, there was nothing on it (for me) because I don't have anxiety*
Structure		Yes: *Exposure, the last part, is important, but I believe it's important and necessary to have the theoretical part before the exposure, so I think it's been very well structured in that sense*
Effect of treatment		
Topic or (sub)theme	Quantitative support	Qualitative support
Affect regulation	*Yes:* ALS-18 (*n =* 8) pre 1.47 (0.31), post 1.09 (0.28) Z = -2.53, *r =* -0.89, *p =* 0.012 *No:* DERS-16 (*n =* 8) pre 3.19 (0.84), post 2.98 (0.96) Z = -0.84, *r =* -0.30, *p =* 0.400	Yes: *Emotions are not dangerous. It's natural. Everyone has emotions. Maybe mine are a bit stronger, but... It's not actually dangerous to feel things*
Functioning	*Yes:* CORE Functioning (*n =* 8) pre 1.47 (0.55), post 1.03 (0.51) Z = -2.10, *r =* -0.74, *p =* 0.035	Yes: *Before, I missed a lot of work because of anxiety. Now, I go to work as I am meant to*
Well-being	*Yes:* WHO-5 (*n =* 8) pre 12.36 (4.17), post 16.38 (3.85) Z = 2.18, *r =* 0.77, *p =* 0.030 *Yes:* CORE Well-being (*n =* 8) pre 2.34 (0.80), post 1.44 (0.61) Z = -2.25, *r =* -0.80, *p =* 0.024	No relevant statements
Anxiety	*No*: GAD-7 (*n* = 8) 10.88 (4.32), post 7.76 (4.13) Z = -1.69, *r =* -0.60, *p =* 0.091	Yes: *I have a bit less anxiety when I move around the city, which was a big issue in the beginning*
Depression	*No*: QIDS (*n =* 8) pre 13.90 (4.55), post 11.86 (4.70) Z = -0.85, *r =* -0.30, *p =* 0.395	No relevant statements
General symptoms	*No:* CORE Global distress (*n =* 8) pre 1.51 (0.45), post 1.07 (0.45) Z = -1.82, *r =* -0.64, *p =* 0.068	No relevant statements

ALS-18, Affective Lability Scale – 18 item version; CSQ-8, Client Satisfaction Questionnaire – 8 item version; CORE-OM, Clinical Outcomes in Routine Evaluation – Outcome Measure; DERS-16, Difficulties in Emotion Regulation Scale – 16 item version; GAD-7, Generalized Anxiety Disorder – 7 item scale; QIDS, Quick Inventory of Depressive Symptomatology; WHO-5, World Health Organization – 5 item Well-being Index.

### 3.3 Qualitative analysis of initial themes

To address the research questions, we focused our TA on responses related to how the participants with BD experienced the UP treatment, particularly regarding the group format. In doing so we identified three main themes: “importance of structure and relevance of content”, “the group as a source for support and learning”, and “skills for understanding and regulating emotions”.

### 3.3 Mixed methods results

The main themes “importance of structure and relevance of content” and “the group as a source for support and learning” were evaluated to align with aspects of acceptability. For the mixed analysis these themes were viewed together with the quantitative findings from the CSQ-8.

The third main theme “skills for understanding and regulating emotions” was evaluated to cover possible effects of the UP treatment. To align this theme with measures of effect more closely, a new thorough review of all participant statements coded within this theme was conducted. This resulted in a subdivision into two subthemes which we called: “effects on emotion regulation” and “changes experienced in daily functioning”. The first subtheme was evaluated to align with measures of emotion regulation and affect lability, whereas the latter subtheme was related to functioning. The complete analysis is presented as a joint display in **Table 2**.

#### 3.3.1 Acceptability: structure, content and group process

Overall, the treatment appeared to be well-accepted by all participants, despite having different views on certain elements when probed for constructive criticism.

##### 3.3.1.1 Importance of structure and relevance of content

Statements in this theme were related to how participants described the specific aspects of the treatment. Statements were coded into this theme if they made some sort of evaluation. We found that these evaluations spoke to the way the treatment was structured, whether the specific content was relevant to how they experienced their problems, or a mixture of both.

Most participants expressed that the content was easy to understand. The structure of the intervention was highlighted as helpful and made the participants feel that they were taken seriously. *“It was understandable. We went through it very systematically”* one participant said. Another commented that the treatment *“(…) felt very structured and professional”*, whereas a third noted how the structure was important in breaking down (his) initial resistance:

*I have a fundamental mistrust of systems, forms, and a schematic, Excel-like understanding of people (…) But then I saw that what the handout forms allow for … is conversations between people who need to talk (…) building blocks we can use so it’s not just floating in an endless conversation of words; rather, ‘okay, this is something we can actually do. It’s concrete, and we can relate to it.’*

The relevance of emotional exposure, which was described as a very important part of the treatment by most participants, was also tied to the structure of the treatment by one participant, noting: *“(…) exposure, the last part, is important. (…) but I believe it’s important and necessary to have the theoretical part before the exposure, so I think it’s been very well structured in that sense”.* Although all participants had favorable things to say about the exposure element, one participant noted how it was a challenge to find a specific focus for exposures: *“(…) for many of the others, it seemed like there were maybe several things that challenged them a bit in their daily lives which were somewhat consistently limiting (…) I found it kind of difficult to identify a problem, like 'okay, now I should address this'“*.

Some participants noted that the content related to anxiety was less relevant for them: *“for my own part, as I also talked about with some other course participants, anxiety is not what bothers me the most. So, I didn’t get much out of that part”* one participant said. Another participant said: *“That list they had made with suggestions for exposures, there was nothing on it because I don’t have anxiety. It doesn’t manifest in that way for me”.* One participant experienced hypomania during the intervention and experienced anxiety exposure as less relevant in that state: *“I did the exercises and all, but I didn’t experience any anxiety from it. I have very little anxiety [during hypomania]. So, the outcome wasn’t what it could have been”.* However, others highlighted the breadth and flexibility of the intervention as useful: *“And then it’s a bit like I said, that I think it’s been nice that you’re not tied to just one thing. Just seeing that (…) it’s emotions in general. It doesn’t have to be just anxiety”*, said one participant.

The handout material received mostly neutral to positive comments, but one participant found that the amount was problematic: *“When there’s too much, it’s hard to extract the essentials (…) from the information given … the message within the information”* and another participant described the amount of handout material as *“overwhelming”.* Somewhat related to these comments was another participant’s remark regarding time: *“they [the therapists] were very kind and understanding, but the only thing … I wish there was a bit more time … It’s set for two hours, but I think it could have been two and a half hours. Because when we were talking about how we did the exposure tasks from last time and so on, it was like … maybe only two or three people had the chance to respond before we had to move on”.*

Homework was accepted and appreciated by all participants, and the amount given was described as appropriate and manageable. However, some noted that they struggled to find appropriate assignments and one participant said: *“I was kind of dependent on having someone to do it with me. (…) So there were some weeks that were harder than others”.* But most participants commented how the homework generally was positive:

*A bit of a push, and the homework assignments, because then I felt that I didn’t have to just talk about it. Next time I come, I have to have done it (…) for me, it was a bit nice to feel that pressure, because it’s so easy to be like: ‘I can’t be bothered today.’*

*Having a goal for the next time means you have to do it. And it’s a bit reassuring that there are three people closely following along. They know what they’re doing too.*

In addition to emotional exposure, the different UP skills were accepted by most participants, with the mindful awareness exercise “three-point check” being most frequently mentioned, along with the skill “cognitive flexibility”.

##### 3.3.1.2 The group as a source for support and learning

This theme was comprised of coded statements related to how peers in the group, or the group format, impacted their experience of the treatment. Statements in this theme were all somehow addressing how the group was a source that helped them feel supported, or they highlighted implicitly or explicitly how the group format allowed for a type of learning that would otherwise not be possible.

All participants spoke favorably about the group format. Feeling safe, welcome and a sense of togetherness was frequently mentioned by participants.

*It’s effective because you gain a sense of community. You’re not alone; you have a place to show up, and it drives your process. You don’t have a responsibility, but … you have a community of love … There’s care and understanding from people who … when you arrive there, you feel like you’re coming home, because you’re in a space where they take you seriously, they don’t patronize you, they don’t look down on you, they don’t judge you—nothing. And that makes you feel very calm.*

This importance of the group was underscored by another participant who noted that *“the first session could have had a slightly longer introduction round. Because the sense of unity in the group turned out to be quite important”*. In line with this, another participant commented how the group developed over the first sessions: *“It was like there was a heavy energy in the whole room. (…) I noticed it especially after, yes, maybe the third or fourth session, that’s when I started to feel like I could relax a bit here”.* One participant noted how the low attendance in session 4 changed the dynamics in a positive way: *“When we were in a smaller group, it became a bit more intense. And I remember the therapists saying at the end, like, 'Yeah, this was a bit of an exhausting session,' or something like that. And I thought, ‘Yeah, this was a bit exhausting, but it was actually good.’ Because it became a bit more intense. It’s supposed to be like that. It’s not supposed to be easy”*.

Getting to know the other group participants and feeling understood for their personal struggle by people who shared many of the same problems was noted as important for several participants.

*I’m very glad that I’ve been in a group. It’s really comforting not to feel alone. And it’s also good to be in such a challenging situation where others understand. Because the people around me, like family and my husband and so on, they’re not going through the same thing.*

One participant also noted how valuable it was to get to know others with BD: *“I don’t know anyone else with bipolar. And then … you hear that the problems you have are the same ones that many others struggle with. So it was really nice to get to know others, talk about it, and feel that it’s not just me”.*

Several comments highlighted the educational aspect of being in a group. One commented *“It was very nice and very educational to work in a group. Because you could relate to others, and many could also relate to what you were saying. How you, in a way, handle emotions”*. Another explained how role-playing a provoking character in the emotional exposure exercise of a fellow participant was helpful: *“It hurts as well, to see yourself in that way … and then to listen to how it feels for the person who is actually subjected to it. (…) But it was very useful to do it”.*

Overall, the favorable statements on acceptance were supported by the CSQ-8 where scores ranged from 26-32, with a mean of 28.78 (SD = 1.99), indicating high satisfaction. Here, seven of the nine participants reported that they were sure they would recommend the treatment to a friend with similar needs, whereas the remaining two thought they would.

#### 3.3.2 Effect of treatment: skills for understanding and regulating emotions

This theme covers many statements related to possible effects of exposure and skill learning. First and foremost, participants talked about using skills to regulate emotions. Secondly, participants explained resulting consequences, such as new behaviors in daily life.

##### 3.3.2.1 Effects on emotion regulation

Numerous statements reflected that the treatment had provided participants with a new and more accepting and non-judgmental perspective on emotions. *“Emotions are not dangerous. It’s natural. Everyone has emotions. Maybe mine are a bit stronger, but … It’s not actually dangerous to feel things”*. One participant explained how an exposure exercise to provoke awkward social attention resulted in useful insight *“I also realized that people paid very little attention to me. The world doesn’t revolve around me. And that was a good experience that gave me a sense of coping”*, whereas another participant experienced particular benefit from understanding and accepting the bodily, physiological nature of emotions, noting; *“The most important thing … well, maybe that I am more capable to handle physical reactions, especially. And to be, not just in relation to anxiety, but to be curious”*.

In addition to gaining a more accepting perspective on emotions, several participants were specific about the use of skills from the treatment, the three-point check in particular. One participant said: *“The thing with the three-point check is about having that kind of non-judgmental perspective (…) I think that mindset has been ingrained in me quite well. It’s come through this process, so it’s kind robust now”*. Another talked about using the technique in specific instances where automatic or rigid thinking was detected: *“I still jump to conclusions quite quickly, which most likely aren’t accurate. But I do a three-point check, and then it actually calms down”.*

The quantitative measures were mixed concerning emotion regulation. The patients´ descriptions of positive experiences aligned well with the significant reduction (*p =* 0.012) in affective lability measured by the ALS-SF pre (M = 1.47, SD = 0.31) to post (M = 1.09, SD = 0.28)) with a large effect size (r *=* -0.89). However, the small change in DERS-16 from pre (M = 3.19, SD = 0.84) to post (M = 2.98, SD = 0.96) intervention was non-significant (p *=* 0.400).

##### 3.3.2.2 Changes experienced in daily functioning

Statements in this subtheme described how new understanding and implementation of skills impacted functioning in different areas of life, particularly social relations. Most changes in functioning concerned social relationships.

*I can receive love. From both friends and romantic relationships, really. It was tough in the beginning, and I still jump to those anxious conclusions all the time. But now, it has calmed down a lot.*

Several participants talked about overcoming barriers to social engagement as an effect of the treatment. One said: *“my overarching goal was to take more social initiative. I invited everyone over to my place for a low-key gathering”*. Another participant noticed a change in how anxiety was handled and interpreted: *“It’s not so much about how I feel directly, but for example, I’ve struggled with, and one thing I worked on was daring to take up* sp*ace in social situations. So … I’ve pushed myself a bit more with that now, thinking that it’s okay. When I feel that anxiety about 'I’ve talked too much,' I can think, ‘It’s not a big deal’”.* Increased openness and lowered threshold for seeking social support was also reported by a participant: *“I am telling people how I feel. I can write a message saying ‘I’ve had a horrible day, let’s do something’. I would never do that before”.*

One participant also noted a change that impacted work functioning: *“Things are a bit easier. It’s a bit easier to get up in the morning and just get started. Before, I missed a lot of work because of anxiety. Now, I go to work as I am meant to”*.

Overall, there seemed to be notable changes in functioning for several participants. This was also supported by the CORE Functioning measure which showed a significant (*p =* 0.035) decrease from pre (M *=* 1.47, SD = 0.55) to post (M = 1.03, SD = 0.51) intervention with a large effect size (*r =* 0.74).

## 4 Discussion

This study explored the feasibility of the UP in group format for a naturalistic sample of patients early in the course of BD in a specialized treatment unit. Using both qualitative and quantitative data, the main purpose was to get information which can serve to adapt, modify and improve the intervention in preparation for a larger controlled trial. Our main finding was that the treatment was feasible to administer with a 100% retention rate, and that participants overall seemed to accept the treatment. Reports on treatment effects suggested that participants experienced improved emotion regulation and functioning. All differences in mean pre- and post-intervention outcome measures were in the expected direction towards reduced symptoms and improved functioning and well-being. The patient-reported changes in social functioning are a possible downstream effect of improved emotion regulation and reduced avoidance, as participants used various UP skills to overcome barriers to interpersonal engagement. However, only measures of functioning, well-being and one measure related to emotion regulation showed a significant change. The small sample size renders the quantitative results susceptible to chance variability, but we here use a mixed-methods approach to validate the results. We also provide some ideas for further development of the intervention.

In the interviews, the participants expressed that they found the intervention acceptable, and the quantitative rating of satisfaction from CSQ-8 with a mean of 28.78 (SD = 1.99) suggests high satisfaction with the treatment. For comparison, this is markedly higher than what has been reported from a screening of general psychiatric services provided in Norway of 23.67 (SD = 6.08) (40), and on par with the mean found in the previous UP for BD study of 28.33 (SD = 3.57) (18).

The pilot feasibility study evaluating the UP with an individual format for BD had 18 sessions (18). It is likely that added sessions and/or time could be valuable in promoting depth of the processing with newly acquired skills. It could also have provided more time to reflect on exposures early in the intervention period. Because this was a group with BD and more complex clinical presentations compared with what has previously been studied in the UP group format (20), more time to understand and implement may be needed for some patients. The strict schedule where all sessions are equally long could also perhaps be more flexible to better adapt to the content of the modules, e.g. longer sessions initially to promote group cohesion and learning of new skills.

Another difference from the previous UP for BD study and our current study is the proportion of participants with BD I vs. BD II. In our study, the majority (67%) had a BD II diagnosis, whereas in the previously published study only 8% had a BD II diagnosis, and the remaining participants had BD I (18). Previous research has shown that affective lability is more prominent in BD II compared to BD I and schizophrenia, and that high affective lability is associated with higher symptom level and reduced social functioning in this population (11). Despite the small sample size, the significant reduction in affective lability observed in this study is promising and suggests that the further exploration of the UP group format as a therapeutic intervention to target affective lability in BD is warranted.

Another noteworthy difference from the previous UP for BD study was a comorbid anxiety disorder as an inclusion criterion (18). In our study more than half (56%) of the participants did not have a comorbid anxiety disorder. We did not specifically adapt the content of the intervention in advance to make the content more relevant for this subgroup, although the therapists did develop individualized plans for each participant. It is notable that the UP group format was found to be so acceptable and useful despite not being tailored to the specific clinical presentations of BD. This is in line with transdiagnostic rationale of the UP, aiming to facilitate broad emotion regulation skills rather than management of disorder-specific symptoms. Though the material generally was well accepted, a larger focus on depression in the BD population may be warranted.

Participants had several clear statements where they described increased functioning, which was supported by the quantitative measure. Though it was not directly stated by the participants, they seemed to describe an attitude where they were actively countering avoidance in everyday life, which was the skill taught in module 5 and used actively in the emotional exposures. Because all participants experienced strong peer support from the group, this attitude could perhaps be strengthened further if given more time to process and share in the group.

This study has several limitations. Due to the small sample size and no control group, the interpretation of findings, especially regarding treatment effects, must be done with caution. Furthermore, we did not collect data on medication use during the intervention, and participants were not restricted from receiving additional psychotherapy during the intervention. However, generalizability was beyond the scope of our focus for this study, and the interpretations are meant to be exploratory in the service of further development.

## 5 Conclusion

Taken together, our results indicate that the UP in group format is a feasible treatment for patients with early BD with varying degrees of comorbidities. The study provides information on how the intervention can be further improved and targeted to meet the needs of this population. Due to the severity of the illness and heterogeneity of the population, both lengthening the duration of treatment sessions and increasing the number of sessions should be considered. An increased focus on depressive symptoms relative to anxiety could increase acceptability. A reduction of written handout material and theoretical presentation should be considered and would provide more time for the implementation and practicing of skills.

## Data Availability

The raw data supporting the conclusions of this article will be made available by the authors, without undue reservation.
